# Multicenter study of plastic vs. self-expanding metal stents in endoscopic ultrasound-guided drainage of walled-off pancreatic necrosis – PROMETHEUS: a randomized controlled trial protocol

**DOI:** 10.1186/s13063-019-3988-x

**Published:** 2019-12-30

**Authors:** Joan B. Gornals, Manuel Perez-Miranda, Enrique Vazquez-Sequeiros, Juan Vila, José M. Esteban, Ferran Gonzalez-Huix, Carlos Guarner-Argente, Andres Sanchez-Yague, Alvaro Teran, Francesc Bas-Cutrina, Carlos De La Serna, Ana Garcia De Paredes, Raquel Ballester, Julio Velasquez-Rodriguez, Silvia Salord, Cristian Tebe, Pilar Hereu, Sebas Videla, On behalf of the Spanish Working Group on Pancreatic Collection Therapy

**Affiliations:** 1 Endoscopy Unit, Department of Digestive Diseases, Hospital Universitari de Bellvitge, Bellvitge Biomedical Research Institute (IDIBELL), University of Barcelona, Feixa Llarga s/n, 08907 L’Hospitalet de Llobregat, Barcelona, Catalonia Spain; 2 Health Sciences, Universitat Oberta de Catalunya, Barcelona, Spain; 3 Endoscopy Unit, Hospital Universitario Rio Hortega, Valladolid, Spain; 4 Endoscopy Unit, Gastroenterology and Hepatology Service, Hospital Ramon y Cajal, IRYCIS, Madrid, Spain; 5 Endoscopy Unit, Complejo Hospitalario de Navarra, Pamplona, Spain; 6 Endoscopy Unit, Department of Digestive Diseases, Hospital Clínico San Carlos, Madrid, Spain; 7 Endoscopy Unit, Hospital Arnau de Vilanova, Lleida, Spain; 8 Endoscopy Unit, Hospital Sant Pau i Santa Creu, Barcelona, Spain; 9 Endoscopy Unit, Hospital Costa del Sol, Marbella, Spain; 10 Department of Digestive Diseases, Hospital Universitario Marqués de Valdecilla, Santander, Spain; 11 Hepato-Bilio-Pancreatic Unit, Department of Digestive Diseases, Hospital Universitari de Bellvitge, Bellvitge Biomedical Research Institute (IDIBELL), University of Barcelona, Barcelona, Spain; 12 Biostatistics Unit, Institute of Biomedical Research of Bellvitge, L’Hospitalet de Llobregat, Barcelona, Spain; 13 Clinical Research and Clinical Trial Unit (UICEC IDIBELL), Plataforma SCRen, Clinical Pharmacology Department, Hospital Universitari de Bellvitge, Bellvitge Biomedical Research Institute (IDIBELL), University of Barcelona, Barcelona, Catalonia Spain

**Keywords:** Endoscopic ultrasound, Lumen-apposing metal stent, Metal self-expanding stent, Plastic stent, Trial, Walled-off necrosis, self-expanding metal stent, Randomized clinical trial

## Abstract

**Background:**

It seems that lumen-apposing metal stents (LAMS) are displacing plastic stents in the therapy of pancreatic-fluid collection in walled-off necrosis (WON). To date, there is no quality of evidence to recommend LAMS as the standard treatment in the management of WON. The theoretical benefit of LAMS over plastic stents needs to be proven.

**Methods/design:**

This is a randomized controlled, multicenter, prospective clinical trial with two parallel groups, without masking. One-hundred and fourteen patients with WON will undergo endoscopic ultrasound (EUS)-guided transmural draining in nine tertiary hospitals in Spain and will be randomized to the LAMS or plastic-stent group. The primary endpoint is the short-term (4 weeks) clinical success determined by the reduction of the collection (to < 50% or < 5 cm in size), along with clinical improvement. Secondary endpoints: long-term (4 months) clinical success (total resolution or 5 cm), procedure duration, level of difficulty, safety, and recurrences.

**Discussion:**

The PROMETHEUS trial has been designed to determine whether LAMS are superior to plastic stents in EUS-guided transmural drainage of WON.

**Trial registration:**

ClinicalTrials.gov, ID: NCT03100578. Registered on 4 April 2017. https://clinicaltrials.gov/ct2/home

## Background

The Atlanta classification of pancreatic-fluid collection establishes that walled-off necrosis (WON) consists of a variable amount of necrotic tissue within a reactive-tissue wall. This derives from the encapsulation of acute necrotic collections. Imaging tests reveal a well-defined wall surrounding the collection, the formation of which typically occurs at about 4 week from the origin of the acute or chronic pancreatitis. The presence of more necrosis worsens the prognosis; mortality in patients with necrotizing pancreatitis is as high as 15%, and it can reach 30% in patients with infected necrosis [1, 2]. In case of symptomatic WON, drainage is considered, and in the last two decades minimally invasive approaches (surgical or endoscopic) have enjoyed increased acceptance in the management of necrotic collections over open surgical necrosectomy, with fewer adverse events (AEs) and less long-term morbidity. Recent trials have shown that endoscopic approaches offer better clinical outcomes, with improvement in quality of life and lower costs [3–6].

In the last few decade, endoscopic techniques, such as the use of endoscopic ultrasound-guided transmural drainage, have been increasingly used as a first-line option for the treatment of symptomatic WON [5, 7]. To date, plastic stents (such as double plastic stents) have been become the mainstay of endoscopic therapy for WON, until the introduction of lumen-apposing metal stents (LAMS) [8–10]. There is the hypothesis that the large diameter of LAMS improves the drainage of WON with respect to plastic stents (7–10 Fr) and carries less time-consuming interventions, with a smaller number of endoscopic procedures to achieve final success. Regarding safety, the risk of leakage, perforation, or migration would theoretically be reduced using the specific stent design of LAMS. However, LAMS are more expensive, and their safety is controversial, with a significantly higher rate of AEs, such as bleeding, compared with plastic stents [11–14] (Table 1).

Although some international systematic endoscopic reviews and meta-analyses have recommended LAMS as the care standard for WON drainage, the quality of evidence is not high (retrospective, registry-based, or non-comparative) that LAMS are superior to PLASTIC stents in the management of WON by EUS-guided transmural drainage, and few studies have compared LAMS and plastic stents [15–20]. For all these reasons, a multicenter, prospective, parallel-group, randomized controlled clinical trial was designed to assess whether LAMS are superior to plastic stents in the endoscopic treatment of WON.

## Methods/design

The PROMETHEUS trial is a randomized controlled, multicenter, prospective clinical trial with two parallel groups, without masking and with a 1:1 allocation ratio. One hundred and fourteen patients with WON will be scheduled for endoscopic ultrasound (EUS)-guided transmural drainage in nine tertiary Spanish hospitals and will be randomized to the LAMS and plastic-stent groups. All the centers are members of the Spanish Society of Digestive Endoscopy (SEED), which acts as the promoter of this trial.

Central ethical approval of the study protocol has been confirmed from the Comité Ético de Investigación Clínica (CEIC) del Hospital Universitari de Bellvitge-IDIBELL (ref approval no. 140/15) and we will not begin recruiting at other centers in the trial until local ethical approval has been obtained. A Standard Protocol Items: Recommendations for Interventional Trials (SPIRIT) Checklist is attached as Additional file 1.

**Table 1:** Comparative table of the two kinds of stents used in transmural drainage of pancreatic collections

Lumen-apposing metal stent		Plastic	
Advantages	Disadvantages	Advantages	Disadvantages
Easy release	Expensive	Economical	Smaller diameter
Wide diameter	Less scientific evidence	Easy extraction	Shorter patency
Better drainage of solid waste and necrosis	Temporary placement	May be left permanently in place	High occlusion rate
Direct necrosectomy via stent	Traumatism caused by the ends	Greater experience (studies)	A single stent is not enough for WON
Longer patency	Not known whether permanent placement is possible	High success rate (> 80–90%, all types of collections)	Multiple > demanding technique (MTGT for WOPN)
Correct visibility			Worse visibility (fluoroscopy)
Short therapeutic time			
Hemostatic effect			
Prevents migration			Leakage of liquid (in ostomy)
Anchoring effect			
Prevents liquid leakage			Migration
Easy extraction			

*MTGT* Multiple transluminal gateway technique, *WOPN* Walled-off pancreatic necrosis

### Study population

This clinical trial will be performed at the endoscopy unit of the digestive diseases departments of nine tertiary and university centers in Spain. In order to participate in this study, the patient must be a candidate for guided transmural drainage with EUS of WON-type pancreatic collection as a local complication in acute pancreatitis. The investigator at each center will be contacted to evaluate the inclusion of the patient in the study. The patient will be correctly informed by personnel knowledgeable about the specifics of the study, who will help to resolve any questions that may arise. The informed consent form will be signed in the presence of participating personnel knowledgeable about the study. The patient has the right to opt out of the study at any time.

The inclusion and exclusion criteria are listed in Table 2.

**Table 2:** Selection criteria for WON walled-off necrosis. ^a^Diagnosis of WON based on imaging procedures

Inclusion criteria:	
*Patients eligible for the trial must comply with all of the following at randomization* • Age 18 years or older • Patient with indication (ASGE, Jacobson BC, GIE2005) of drainage of only one type^a^ related to the symptomatology, as a local complication of previous acute pancreatitis • Patient capable of understanding and signing informed consent form • Patient understanding the type of study and complying with the follow-up of complementary tests during the study’s duration	
Exclusion criteria	
• Pregnancy or breast-feeding • Severe coagulation disorder: INR > 1.5 not correctible with administration of plasma and/or platelets < 50,000/mm^3^ • Asymptomatic patients, without clinical indication of drainage, except for those with vascular compression involvement • Non-identification of solid content during EUS procedure • Failure to sign informed consent form • Patients with intellectual handicap who are unable to understand the nature and possible consequences of the study, unless there is a competent legal representative • Patients unable to adhere to subsequent follow-up requirements • Conditions that preclude upper digestive endoscopy, such as stenosis	

*Note*: If there are several pancreatic collections, this does not exclude the patient from the trial. The patient is only excluded if there is more than one symptomatic collection to be drained

*EUS* endoscopic ultrasound, *INR* international normalized ratio

### Recruitment

Principal investigators from each center will have the task of presenting strategies to promote enrollment and to ensure the target sample size.

### Randomization and masking

Patients will be enrolled in this trial by gastroenterologists, surgeons, and endoscopists who will evaluate the cases in the inpatient wards or in the outpatient consultation areas. The participants will be randomized with a random-number table generated by an online platform.

A code list will be generated by randomization with a 1:1 randomization ratio, by blocks, stratified by centers and by American Society of Anesthesiologists’ classification (ASA) score. Each individual will be assigned a randomization code along with the treatment that corresponds. Once the patient meets the eligibility criteria and has provided informed consent, we will proceed to the allocation of each participant centrally, ensuring allocation concealment, and based on the randomization list. To prevent different subject recruitment rates at the various hospitals from interfering with the development of the study, the entire population will be randomized in blocks of four between the two treatment possibilities.

### Procedural technique

#### Qualification of centers

The participation of a minimum of nine hospitals with an inclusion of about 13–15 patients per year per hospital is required. The investigators at the participating centers will all have experience in endoscopic intervention and therapeutics, previous experience in EUS-guided transmural drainage with metal and plastic stents (> 25 overall), and a minimum of 10 procedures per year, in addition to appropriate material at their disposal for carrying out transmural drainage with both types of stent.

#### General description of the technique

Each selected case will ensure a conclusive diagnostic revision with WON. In case of doubt, EUS-guided, fine-needle aspiration will be considered prior to drainage to rule out cystic tumor. Prophylactic antibiotic therapy will be administered in accordance with the protocol of each center. In case of INR > 1.5, this will be corrected in accordance with the protocol of each center until INR < 1.5. Procedures will be performed under deep sedation in accordance with the directives of each center. In case of collection > 10 cm (> 700 mL), tracheal intubation by anesthesiologist is recommended. Carbon dioxide insufflation is recommended, especially for endoscopic necrosectomy. The WON will be localized by using a linear echoendoscope, selecting the appropriate optimal region for carrying out the EUS-guided puncture. The collection will be punctured with a 19-G needle (plastic or LAMS group) or directly with an electrocauterizing device (freehand technique, only in the LAMS group). The guidewire will be advanced and coiled into the WON cavity. Then, the transmural ostomy will be carried out in accordance with the normal procedure of the experienced endoscopist. The use of a fluoroscope will be decided upon on the basis of technical considerations and the opinion of the endoscopic interventionist. The use (or not) of a fluoroscope will be noted in the case report form (CRF). The scale of the ostomy will be determined by the size of the collection (see Table 3).

**Table 3:** Number of stents along with technical variations depending on size of WON, observed with EUS during procedure

Type	LAMS (*n*, ø)	Plastic (*n*, ø)
WON < 10 cm	1, 10–15–20 mm	≥ 1, minimum 1 of 10Fr (+ ostomy 8–10 mm)
WON > 10 cm	≥ 1, > 15 mm	≥ 2, minimum 1 of 10Fr (+ ostomy 10–15 mm)

*LAMS* lumen-apposing metal stent; *WON* walled-off necrosis, *FR* French

After the interventional procedure, all inpatients cases will be returned to the hospital ward and will discharged after clinical improvement. Outpatients will spend a minimal of 24 h under observation and will be discharged the next day unless there is no improvement of symptoms or the appearance of AEs.

#### Plastic-stent group

Double-pigtail plastic stents (5–10 cm in length, diameter 7–8.5 10 French gauge (Fr), Advanix, Boston Scientific, Spencer, USA) will be used. A minimum of one 10Fr pigtail plastic stent will be placed. After initial EUS-guided access, the ostomy will be dilated first, using a cystotome, and secondly with a balloon dilation. The plastic stent will be inserted and delivered following the routine technique of each interventional endoscopist. The number of the plastic stent and size of the balloon used to dilate the ostomy will depend on the WON size and content (see Table 3). The time to plastic stent withdrawal will be considered until total resolution by imaging. If there is pancreatic-duct involvement, non-withdrawal of stent will be considered.

#### Metal group

The metal stents used in this study will be LAMS (10, 15, or 20 mm in diameter, and 10 mm in length, HotAXIOS stent with an electrocautery-enhanced delivery system, Boston Scientific, Madrid, Spain). This is a self-expanding metal stent totally covered with luminal apposition. After the EUS-guided access into the WON using first a 19-G or directly with the electrocautery tip, the delivery system will be advanced into the cavity and the distal flange will be deployed under EUS guidance. Otherwise, the proximal flange will be released under EUS or endoscopic guidance. The time to stent withdrawal will depend on total resolution by imaging. However, there will be the intention to remove a LAMS no later than 4–6 weeks.

#### Additional interventions

Necrosectomy will be considered in WON with predominantly solid debris, using the technique at the discretion of the endoscopist. In cases that require sessions of endoscopic necrosectomy, the different technical variants described in the literature will be used (irrigation technique with normal saline; mechanical technique using the common devices as snares, Dormia baskets or retrieval nets for extraction of necrotic debris; or combined with nasocystic catheters). The periodicity will be every 2–5 days depending on the decision of the expert endoscopist and the clinical evolution of the patient.

#### Additional comments

In the event of technical failure in the placement of the stent (plastic or LAMS) for any reason, alternative treatment will be decided upon in accordance with the directives of the endoscopic interventionist, with the aim of offering the patient the best possible solution.

The first controls will be on the first day following and at 4 weeks (see Table 4). If there is no short-term clinical success, the most beneficial therapeutic approach for the patient will be adopted in accordance with the criteria of the patient’s medical team (for example: the use of technical variants at initial drainage such as placement of coaxial plastic pigtail within the LAMS, replacement or change the stent type, use of nasocystic drainage or other known variants).

**Table 4:** Data management, calendar

Timepoint/stages	Study Period									
	Baseline	Intervention	24 h	7 days ±	4 weeks ± 3 days	8 weeks ± 5 days (Telephone)	16 weeks (4 months) ± 10 days	6 months ± 10 days (Telephone)	8 months ± 10 days	12 months ± 10 days (Telephone)
Enrollment										
Informed consent	X									
Clinical history and exploration	X									
Eligibility screen	X									
Allocation	X									
Intervention										
Implantation		X								
Removal					X					
INR		X								
Assessments:										
Blood test	X		X		X		X		X	
Imaging test	X	X			X	X^a^	X		X	
Symptomatology	X		X	X	X	X	X	X	X	X
Visit			X		X		X		X	
Telephone contact				X		X		X		X
Adverse effects	X	X	X	X	X	X	X	X	X	X
Primary outcome					X					
Secondary outcomes					X					
Medication	X	X	X	X	X	X	X	X	X	X

^a^Only in case of radiological clinical success but with persistence of the collection > 5 cm

Every intervention, diagnostic procedure, and additional therapeutic contribution will be noted in the CRF.

In the case of collections of significant size (i.e., 14 cm) with clinical-radiological success but without the disappearance of the collection (i.e., CTMD reduction from 14 to 6 cm), precluding the removal of the stent, a new CTMD will be performed at 4 weeks (8 weeks) to assess removal of the stent.

Therefore, the removal of a stent is contemplated when there is disappearance of the collection or a decrease < 5 cm.

Patients will remain in the hospital for a minimum of 24 h for clinical observation, under medical supervision.

### Clinical evaluation and follow-up

#### Data collection

The collecting of clinical information on the patients will begin at the outset (baseline) and will continue with follow-up as established and defined in the study. AEs will be noted from the beginning of the test until the conclusion of follow-up by means of scheduled controls.

#### Calendar

The following time-points and data items will constitute the data collection from the beginning through successive controls: The collecting of data for purposes of documentation will be carried out using a CRF, which will serve as an easily accessed source of information. After collection, the data will be introduced into an electronic database by the participating investigator of each center.

#### Follow-up

Patients will be assessed (visit, telephone call) on days 1 and 7, at 4, 8, and 16 weeks, and at 6, 8, and 12 months by personnel participating in the study, with the aim of obtaining information regarding signs and symptoms pointing to possible stent obstruction or migration, recurrence, or other AEs.

Any instances of death during the follow-up will be investigated to rule out any possible relation to the endoscopic procedure. Such occurrences will also be recorded in the CRF.

If there is clinical suspicion of obstruction or migration of the stent, an upper endoscopy will be carried out. Based on the findings of this procedure, the problem will be resolved in accordance with the directives of the intervening endoscopist. Any additional procedure or endoscopic intervention will be duly documented.

Complications will be handled and treated in accordance with the directives of the patient’s medical team. All additional tests and interventions will be duly documented.

### Definitions

The term WON is in accordance with the literature (latest revision of Atlanta, Banks PA et al. Gut 2013) [1]. At least two imaging tests will be required (CTMD, MRI, EUS) prior to the transmural drainage, the results of which must be in agreement on the classification of the collection as WON.

Technical success is defined as the correct release of the stent at both ends, with observed drainage of the liquid.

Clinical success is defined as the significant reduction of the collection along with clinical resolution.

Recurrence is defined as a symptomatic pancreatic collection diagnosed with imaging test during the follow-up of prior procedure with initial clinical success.

AEs are defined as undesirable situations suffered by patients during the study, whether related or not to the EUS-guided transmural drainage with a stent (plastic or metal). All AEs referred by patients or observed by the medical team will be duly documented. All serious AEs will be detailed, and the study promoter/principal investigator will be notified within 7 days. In the event of a death, notification will be made within 24 h. AEs will be recorded in both the clinical history of the patient and the CRF, with appropriate medical terminology. Whenever possible, the diagnosis rather than the symptoms will be recorded. These guidelines are to be followed from the time of the signing of informed consent until 30 days after the final visit in the study calendar. AEs will be classified as mild, moderate, serious, or fatal, in accordance with the nomenclature for AEs in endoscopy (ASGE Workshops 2010, Cotton GIE 2010; 71:446–54). The determination as to whether an AE is related to the EUS-guided transmural drainage with stent (plastic or metal) will be made by the patient’s medical team, the local investigator, and the principal investigator of the study.

In Additional file 2 is included information about AE definitions and MEDDEV guidelines. “Research product security surveillance” section, according to the definitions set out in the MEDDEV 2.7/3 guidelines (rev 3, May 2015) “Guidelines on medical devices: Clinical investigations: Serious adverse event reporting under Directives 90/385/EECC and 93/42/EEC” (Additional file 2).

### Outcomes

The primary outcome is the short-term (4 weeks) clinical success (metal vs. plastic) determined by the reduction of the collection (to < 50% or < 5 cm in size), along with clinical improvement.

The secondary outcomes are long-term (4 months) clinical success (metal vs. plastic) determined by total resolution or 5 cm, along with clinical improvement. Technical: to assess the duration of the procedure and the level of difficulty. Safety: to assess AE (early and late). Hospital length of stay. Recurrences. Financial: evaluate the relative costs of the two strategies.

### Sample-size calculation

The sample-size calculation is based on the primary hypothesis of detecting statistically significant differences in the percentage of clinical success in the LAMS and plastic-stents groups at 4 weeks after the intervention. Published data suggest that the clinical success rate at 4 weeks in the LAMS group is expected to be 0.75 and in the plastic-stent group it is expected to be 0.5. Fifty-seven patients will be recruited in each group to reject the null hypothesis that the proportion of clinical success in the LAMS group is equal to that of the plastic group with an 80% power. The type-I error associated with this test will be 5%. To evaluate the hypothesis, the chi-square test or Fisher’s exact test will be used depending on of the application criteria. For the calculation, a planned interim analysis will be made for half of the recruitment using O’Brien-Fleming’s type-I error expense function and a global loss rate of 5%. http://www.stat.ubc.ca/~rollin/stats/ssize/b2.html

### Statistical analysis

All study variables will be presented for stent groups and in total, using descriptive statistics consistent with the nature of the variable. The continuous variables will be described indicating the number of non-missing observations, the mean, the standard deviation, the minimum, the first quartile, the median, the third quartile, and the maximum. Categorical variables will be described indicating the number of non-missing observations and the percentages of the different categories by column.

Main analysis: main outcome is the percentage of patients’ radiological (morphological) success between transmural drainage of the collection, measured at 4 weeks from intervention. The null hypothesis suggests that there are no differences between the proportions of the intervention group and the control group.

The level of statistical significance has been set at 5%. To test the hypothesis, use the chi-square test or Fisher’s exact test depending on the application criteria will be used. To quantify the magnitude of the difference, the relative risk of success will be estimated, in the metal-stent group with respect to the plastic-stent group and its confidence interval will be calculated at 95%.

#### Secondary variable analyses

To determine which factors are associated with clinical-radiological success in the short term, a multivariate logistic regression will be carried out. The variables of age and sex, and factors predicting the location of the disease, treatments received, size, and characteristics of the collection, previous ASA, etc. will be taken. In addition, the appearance of clinical recurrence will be considered by means of an analysis of Kaplan-Meier survival. The factors associated with clinical recurrence will be explored through a multivariate Cox proportional risk model and adjustment variables will also be taken.

#### Subgroup analysis

The main analysis will also be carried out in the following subgroups:

ASA patients I–II vs. II–IV.

#### Data management

Throughout the study the promoters will monitor the quality of the trial with special attention to protocol deviations and the quality of the data entered in the database. At the end of the trial, a meeting will be held to consider the data management report. This report will describe the different deviations from the protocol identified in each of the patients. These deviations will be classified as major or minor, and those patients with major deviations will be excluded from the protocol analysis. After the meeting, the suitability of the database for analysis will be considered, and the database will then be closed.

#### Statistical analysis plan

The statistical analysis plan will be finalized before the close of the database. This plan will include all the analysis described and others, mainly on the sensitivity of the results and the management of the missing data. In the event that in the plan of statistical analysis there is some deviation in the analysis of the main variable, an addendum to the protocol will be made. No changes will be made to the original analysis plan once the database is closed.

### Cost analysis

The procedure for determining the cost of the diagnostic test is made up of several steps: calculation of the unit cost, and accounting for all the costs associated with the test, both direct and indirect.

Calculation process: observation of the performance and accounting for all the factors involved in the procedure (units, time, number of professionals involved).

Criteria to be considered: human resources, disposable material, generic fungible supplies, pharmacy, laundry, equipment, repairs/maintenance of equipment and facilities, energy, cleaning, waste handling, rental, telephone (calls to contact subjects in follow-up), structural costs, and hospital admissions.

### Other considerations

#### Rescue

Depending on the initial endoscopic treatment carried out, a cross-over rescue treatment may be considered when the initial protocol treatment fails. For the plastic treatment group ➔ metal stent (LAMS); for the metal treatment group ➔ plastic stent. Another accepted rescue technique (only in cases of initial treatment failure) in the branch of metal stent: insertion of coaxial plastic pigtail within a LAMS.

In all these cases, the follow-up of the patients will be maintained until the end of the study according to protocol. Alternatively, if a cross-over treatment or technical variant is not possible, percutaneous surgical or radiological treatment will be offered.

#### Withdrawal

Any AE or other clinical condition of the patient which, at the clinician’s discretion, warrants withdrawal of the patient from the study; pregnancy; or expressed wishes of the patient. Withdrawal from treatment will not mean suspension of the study, given that follow-up will be maintained until the end of the study in accordance with the protocol.

The need for surgical intervention for: in these cases, patient follow-up will be ended.

### Ethical aspects and confidentiality

The protocol will be approved by the CEIC of each participating hospital as well as that of the coordinating center (HUB). The study researchers will carry out their tasks in compliance with ethical principles of clinical research established in the Declaration of Helsinki, and with the norms of Good Clinical Practices. It is planned to hire a policy to cover the concepts and compensations according to current legislation (RD 1591/2009) that regulates clinical investigation with healthcare products. Before starting the clinical trial, it is planned to request authorization of the Spanish Agency for Medicines and Health Products (AEMPS) and the CEICs. Before inclusion of the patient in the trial, a written informed consent will be requested. In relation to the study data we will follow the provisions of Organic Law 15/1999 of 13 December on “Protection of Personal Data.”

### Publication of results

There is a commitment to publish the results of this study in high-impact international journals, should the results be of sufficient scientific interest. However, no patient names will appear in any article, and no one, with the exception of the researchers in this study and the members of the Hospital Ethical Committees, will have access to the data, in accordance with the Law on the Protection of Data of a Personal Nature.

## Discussion

Although open surgical necrosectomy has been the traditional treatment of choice in patients with infected or symptomatic pancreatic necrosis, other minimally invasive techniques have been developed in recent years (endoscopic necrosectomy, guided radiological percutaneous drainage, and retroperitoneal treatment) for treating collections, so as to improve on the high morbidity and mortality rates of traditional surgical treatment [3, 21–23].

At present, endoscopic transmural drainage plus endoscopic necrosectomy represents a viable technique that is reasonably safe and effective when carried out in centers that have experience in doing so. Although each of these is a minimally invasive endoscopic technique, they are not entirely free of complications, the most common of which are bleeding, perforation, post-procedural infection, and stent migration [7, 24]. Additionally, with the continuous technological advances being made and the appearance of new materials for endoscopic use, doubts have arisen as to which devices are best to use. One clear example of this uncertainty is the choice of stent. To date, most published studies on guided transmural drainage with endoscopy have involved double pigtail plastic stents, with the number and diameter varying depending on the collection type [23, 24]. In the past years, some reports have been published on the use of self-expanding covered metal stents offering greater diameter, and therefore greater volume in the drainage of the collection [25–27]. However, both types of stent are intended for bile drainage and are not expressly designed for transmural drainage of abdominal collections, so more dedicated stents were investigated [8].

Recently, LAMS designed for the drainage of pancreatic collections have appeared with demonstrated efficacy in a number of studies but they are also more costly [16–20]. These stents are totally covered and offer a maximum caliber of 15–20 mm, thereby allowing for endoscopic necrosectomy in repeated sessions without the need for replacement. In our experience, they permit transmural drainage of pancreatic collections and endoscopic necrosectomy if it is needed, they are safe and effective, and they reduce the duration of the procedure [10, 28].

However, with the increasing use of these stents, significant LAMS-related AEs (i.e., severe delayed bleeding, buried stent syndrome, obstruction, migration) have been reported in several papers.

To date there is only one comparative prospective study of self-expanding LAMS type metal stents, versus plastic stents in the endoscopic treatment of WON-type pancreatic collections, and it concluded that there were no significant differences in treatment outcomes between the two. In order to minimize LAMS-related AEs, they recommend follow-up imaging and LAMS removal at 3 weeks if collection is resolved [29].

The PROMETHEUS trial is supported by the Spanish Society of Digestive Endoscopy and includes nine tertiary centers, as well as experts in the management of WON. Hospital Universitari de Bellvitge has the leadership and main role in centralizing the decisions in case of doubts and controversies, and in limiting heterogeneity.

In conclusion, this randomized multicenter trial is necessary and essential in the effort to clarify the safety and theoretical superiority of LAMS in the management of WON, compared with plastic stents.

### Trial status

*Protocol of submitted version, number and date*: number 2.1; date June 2018

*Recruitment*: Start date 27 June 2017 and recruitment will be completed by June 2020

*Revision chronology*:

a- *PROMETHEUS, June 2017, original: version 1,* first draft of the study protocol.
b- *PROMETHEUS, September 2017, amendment n° 1: version 2.*
  Main amendments: (1) to clarify the second inclusion criterion – in case of more than one collection, the EUS-guided drainage will be limited to *only one* pancreatic collection (WON), related to the symptomatology (Table 2); (2) addition of the “Research product security surveillance” section, according to the definitions set out in the MEDDEV 2.7/3 guidelines (rev 3, May 2015) “Guidelines on medical devices: Clinical investigations: Serious Adverse event reporting under Directives 90/385/EECC and 93/42/EEC” (Additional file 2); (3) extension to nine centers with respect to the initial protocol (four centers); (4) procedural technique, rescue section: in case of failure, adding of the possibility of a “Technical variant” in LAMS group – an insertion of coaxial plastic pigtail within the metal stent
  Additional changes: (1) addition of new and relevant references; change of the grading of the AE classification (immediate, early, and late); (2) commercial name of each stent; (3) start delayed, from the beginning of 2016 to June 2017; (4) procedural technique section: additional comments regarding when and how some technical variants can be made; and technical notes about necrosectomy technique; (5) data management paragraph in the statistical section; and (6) telephone call at 7 days, and additional new legend explaining the possibility of a second imaging procedure at 8 weeks, only in case of radiological clinical success but with persistence of the collection > 5 cm (Table 4, Timeline)
c- *PROMETHEUS, June 2018, amendment n°2: version 2.1 – definitive*
  Minor changes: addition of a new size of LAMS (20 mm in diameter).
  AEMPS and CEIC have been notified after every amendment, with acceptance by each institution

## Supplementary information

**Additional file 1.** Standard Protocol Items: Recommendations for Interventional Trial (SPIRIT) Checklist.

**Additional file 2.** Safety definitions adverse events (AEs).

## Data Availability

Minimal dataset necessary to interpret the findings available from the corresponding author on reasonable request.

## Abbreviations

AE: Adverse event
AEMPS: Spanish Agency for Medicines and Health Products
ASA: American Society of Anesthesiologists’ classification
CEIC: Clinical Research Ethics Committee
CRF: Case report form
CTMD: Computed tomography multidetector
EUS: Endoscopic ultrasound
LAMS: Lumen-apposing metal stent
MRI: Magnetic resonance imaging
SEED: Spanish Society of Digestive Endoscopy
WON: Walled-off necrosis
