# Supplementary material for: Multicenter study of plastic vs. self-expanding metal stents in endoscopic ultrasound-guided drainage of walled-off pancreatic necrosis – PROMETHEUS: a randomized controlled trial protocol
Source: Trials. 2019 Dec 30;20:791. doi: 10.1186/s13063-019-3988-x (PMC6937819; doi:10.1186/s13063-019-3988-x)
Supplement: Supplementary file 2 — Additional file 2. Safety definitions adverse events (AEs). [file 13063_2019_3988_MOESM2_ESM.docx]

**RESEARCH PRODUCT SECURITY SURVEILLANCE**

**Definitions**

The following are based on the MEDDEV 2.7 / 3 guidelines (rev 3, May 2015) "Guidelines on medical devices: Clinical research: Report of serious adverse events according to Directives 90/385 / EECC and 93/42 / EEC", concerning the safety in clinical research with Medical Devices, which is determined according to the causal relationship and / or severity of adverse events:

• Adverse event (AE)

• Severe adverse event (SAE)

• Device deficiency

• Adverse effect of the device (AED)

• Serious adverse effect of the device (SAED)

• Unforeseen serious adverse effect of the device (USAED)

**Adverse Event (AE):**

It is any unwanted medical event, unforeseen illness or injury or adverse clinical sign (including abnormal laboratory results) in subjects, users or other persons, whether or not it is related to the medical device under investigation.

This definition encompasses the events related to the device under investigation and those related to the comparator device.

**Serious Adverse Event (SAE):**

AE that leads to:

- death or permanent injury to a body structure or function

- serious deterioration of the subject's health that:

* leading to a life-threatening illness or injury

* of place or permanent disability of a body structure or body function

* specify hospitalization or prolong an existing hospitalization

* require a medical or surgical intervention to prevent a disease with risk of loss of life.

- produce fetal distress, fetal death or a congenital anomaly or a birth defect.

Any hospitalization previously scheduled for a pre-existing condition or required according to the Protocol, without this causing serious deterioration in the state of health, will not be considered AAG for notification to the Promoter.

**Product Deficiency:**

Inadequacy of a medical device with respect to its identification, quality, durability, reliability, safety or operation.

**Adverse Effect of the Device (AED):**

It is any adverse effect related to the use of a medical device under investigation.

This definition includes any adverse event resulting from insufficient or inappropriate instructions for use, placement, implantation, installation or handling, or any malfunction of the medical device under investigation. The definition also includes any effect caused by user error or intentional misuse of the medical device of the investigation.

**Serious Adverse Effect of the Device (SAED):**

Adverse effect of the Device under investigation that has resulted in any of the characteristic consequences of a Serious Adverse Event.

**Unintended Serious Adverse Effect of the Device (USAED):**

Serious adverse effect of the device under investigation which, due to its nature, incidence, severity or consequences, has not been identified in the updated version of the risk analysis report (in this case, a manual of instructions for use).

**Collection procedure for AEs:**

Researchers will be responsible for documenting all AEs in the patient's medical history based on what the patient refers spontaneously or in response to the investigator's interrogation at each of the visits planned in the study calendar. Likewise, the causal relationship with the product under investigation of the trial will be evaluated and recorded in the clinical history.

If an AE is related or not to the transmural drainage by EUS with stwents (plastic or metal), it will be decided according to the opinion of the Investigating team of the participating center.

The causal relationship for an AA will be established according to the definitions contained in the MEDDEV 2.7 / 3 guidelines (rev 3, May 2015), establishing 5 possible categories as summarized below:

- Not related: when the relationship with the use of the device or procedures can be ruled out (it does not constitute a side effect of the device, there is no temporary relationship with the use of the device, it is not a known response pattern to the use of the device, discontinuation of the use of the device or its reuse does not impact the event or is not expected by the use of the device the affectation of a certain affected organ or body region).

- Unlikely: the relationship with the use of the device is not relevant and / or the event can be explained by another cause, although it is necessary to obtain more information about it.

- Possible: the relationship with the use of the device is weak, but cannot be completely ruled out. There are other alternative causes to the event that are possible.

- Likely: the relationship with the use of the device is relevant and / or the event cannot be reasonably explained by other causes.

- Related: the event is related without any doubt to the use of the device or to the procedures (it is a side effect of the device, there is a temporary relationship with the use of the device, it affects an organ or body region in which it has been used the device, is a known response pattern to the use of the device, the discontinuation of the use of the device or its reuse impacts the event, derives from a misuse of the device or other possible causes have been ruled out).

AEs will be classified as mild, moderate and severe according to the AE nomenclature for endoscopy (ASGE Workshops 2010, Cotton GIE 2010; 71: 446-54). Likewise, AEs will be classified as serious or non-serious according to the definition previously described.

The Investigator will document in clinical course, the AE that the subject presents from the signing of the informed consent until the last visit of the study.

In the eCRF, the AEs related to the Product under Investigation or to the Test Procedures and all AEs, related or not, will be transcribed.

The eCRF will collect the following information: description of the event, start date, end date of the event, whether or not it is related to the product under investigation or to the study procedures, result of the AA, whether it is serious or not and the measures taken.

The measures taken will be classified as: none, home observation, need for hospitalization or other.

The result of the event will be recorded as: event resolved, resolved with sequels, in progress or death.

Examples of **non-serious AEs** (mild-moderate) **related to endoscopy:**

- Pneumoperitoneum not requiring treatment
- Liquid leakage without clinical signs of peritonitis
- Hemorrhaging: non-significant drop in hemoglobin (< 2 mmol/L), not requiring transfusion or re-intervention
- Migration of the stent or movement of the nasocystic catheter, not requiring re-intervention

The evaluation of the “severity” is independent of the causality relationship with the product under investigation and will be determined according to the definition of the MEDDEV 2.7 / 3 guidelines (rev. May 3, 2015).

Examples **of serious AEs** **related to endoscopy:**

- Pneumoperitoneum requiring treatment
- Liquid leakage with clinical signs of peritonitis
- Hemorrhaging: significant drop in hemoglobin (≥2mmol/L), and/or need for transfusion or re-intervention due to bleeding of the ostomy or stent traumatism
- Unscheduled endoscopy or surgical intervention owing to complication
- Migration of the stent or movement of the nasocystic catheter, requiring re-intervention.

Specific AEs that must be reported and included in the CRF:

**Immediate complications related to the procedure (within the first 24 h):**

- - None
  - Bronchoaspiration
  - Respiratory depression Sat O2 < 90%
  - Cardiorespiratory arrest
  - Arrhythmia
  - Allergic reaction
  - Pain
  - Hemorrhaging: self-limiting (spontaneous hemostasis)/requiring endoscopic treatment (sputtering bleeding/profuse bleeding)
  - Perforation: conservative, endoscopic/surgical treatment
  - Exitus
  - Others.

**Earlier (within first 7 days) and late (past 7 days after the procedure):**

- - Pain
  - Hemorrhaging: self-limiting (spontaneous hemostasis)/requiring endoscopic treatment (sputtering bleeding/profuse bleeding)
  - Infection/stent obstruction
  - Migration
  - Perforation: conservative, endoscopic/surgical treatment
  - Exitus
  - Others:

Others: The previously listed AAs will be registered as immediate, early or late complications in visits according to the protocol established in the eCRF. In the event that any of the AE is classified as serious, the notification of said AAG must be carried out as specified below.

**Notification of SAE / SAED / USAED / Product Deficiency**

**Notification from Investigator to Promoter**

To ensure patient safety, each SAE must be notified, regardless of the suspected causality, that occurs after the patient has given informed consent and until the last visit made according to protocol.

This notification must be sent immediately after the researcher is aware of the SAE without it taking more than 3 calendar days.

Recurring episodes, complications, or progression of the initial SAE should be notified as follow-up to the original episode. This notification must be sent immediately without a delay of more than 7 days since the investigator has received the new follow-up information and / or is aware of it.

**Notification of the Promoter to the Competent Authorities:**

According to the guidelines of the MEDDEV 2.7 / 3 guidelines (rev. May 3, 2015), all SAEs, SAED, USAED and deficiencies of the Medical Device that have caused an SAE will be communicated to the Competent Authorities (AEMPS / CEICs) .

In those cases where there is an imminent risk of death, serious injuries or serious illnesses and that require therapeutic measures for the subjects, patients or users of the medical device or carry new findings, reportable events will be notified immediately and not beyond 2 calendar days from the knowledge of the Promoter.

The rest of reportable events will also be notified immediately and within a period not exceeding 7 calendar days from the knowledge of the promoter.

The monitoring of safety data is expected in terms of hemorrhage and obstruction variables that have a significant clinical impact by a Safety Data Monitoring Committee (i.e., that involve the need for transfusion, complementary tests, lengthen hospital stay and optimization or modification of medical treatment, as well as the need for surgical intervention). This Committee will act in accordance with the instructions in the Instruction Manual and may decide whether the protocol modification or premature termination of the study is necessary. The decision of the committee will be communicated to the promoter.
